# Supplementary material for: Hybrid Models and Biological Model Reduction with PyDSTool
Source: PLoS Comput Biol. 2012 Aug 9;8(8):e1002628. doi: 10.1371/journal.pcbi.1002628 (PMC3415397; doi:10.1371/journal.pcbi.1002628)
Supplement: Text S4 — Complete source code for the PyDSTool package (version 0.88.120504). Includes API documentation and help files linking to web pages. This file is identical to the current public release on Sourceforge.net. (ZIP) [file pcbi.1002628.s004.zip › PyDSTool/html/PyDSTool.Toolbox.optimizers.line_search.hyperbolic_line_search-pysrc.html]

xml version="1.0" encoding="ascii"?


PyDSTool.Toolbox.optimizers.line\_search.hyperbolic\_line\_search


| Home | Trees | Indices | Help | PyDSTool |
| --- | --- | --- | --- | --- |

Package PyDSTool ::
Package Toolbox ::
Package optimizers ::
Package line\_search ::
Module hyperbolic\_line\_search

[hide private]

[frames] | no frames]

# Source Code for Module PyDSTool.Toolbox.optimizers.line\_search.hyperbolic\_line\_search

```
 1   
 2  # Matthieu Brucher 
 3  # Last Change : 2007-12-18 09:47 
 4   
 5  """ 
 6  An hyperbolic line search, in fact no searches at all 
 7  """ 
 8   


9 -class HyperbolicLineSearch(object):


10    """ 
11    An inverse line search, takes a point, adds a step (1/(1+iterations)) and returns it 
12    """ 


13 -  def __init__(self, alpha_step = 1., **kwargs):


14      """ 
15      Needs to have : 
16        - nothing 
17      Can have : 
18        - a step modifier, a factor to modulate the step (alpha_step = 1.) 
19      """ 
20      self.stepSize = alpha_step

21   


22 -  def __call__(self, origin, state, **kwargs):


23      """ 
24      Returns a good candidate 
25      Parameters : 
26        - origin is the origin of the search 
27        - state is the state of the optimizer 
28      """ 
29      direction = state['direction'] 
30      if 'initial_alpha_step' in state: 
31        alpha = state['initial_alpha_step'] /( 1 + state['iteration']) 
32        state['alpha_step'] = alpha 
33      else: 
34        alpha = self.stepSize /( 1 + state['iteration']) 
35        state['alpha_step'] = alpha 
36      return origin + alpha * direction

37
```

  


| Home | Trees | Indices | Help | PyDSTool |
| --- | --- | --- | --- | --- |

|  |  |
| --- | --- |
| Generated by Epydoc 3.0.1 on Fri May 4 15:24:12 2012 | http://epydoc.sourceforge.net |
